# Large extents of intensive land use limit community reorganization during climate warming

**DOI:** 10.1111/gcb.13587

**Published:** 2017-01-10

**Authors:** Tom H. Oliver, Simon Gillings, James W. Pearce‐Higgins, Tom Brereton, Humphrey Q. P. Crick, Simon J. Duffield, Michael D. Morecroft, David B. Roy

**Affiliations:** ^1^ School of Biological Sciences University of Reading Whiteknights, Harborne Building Reading RG6 6AS UK; ^2^ NERC Centre for Ecology & Hydrology Maclean Building, Benson Lane, Crowmarsh Gifford Wallingford Oxfordshire OX10 8BB UK; ^3^ British Trust for Ornithology The Nunnery Thetford Norfolk IP24 2PU UK; ^4^ Butterfly Conservation Manor Yard, East Lulworth Wareham Dorset BH20 5QP UK; ^5^ Natural England Eastbrook, Shaftesbury Rd Cambridge CB2 8DR UK

**Keywords:** climate change impacts, climate change indicators, community shifts, community temperature index, land use–climate interactions, land‐use impacts, land‐use intensity

## Abstract

Climate change is increasingly altering the composition of ecological communities, in combination with other environmental pressures such as high‐intensity land use. Pressures are expected to interact in their effects, but the extent to which intensive human land use constrains community responses to climate change is currently unclear. A generic indicator of climate change impact, the community temperature index (CTI), has previously been used to suggest that both bird and butterflies are successfully ‘tracking’ climate change. Here, we assessed community changes at over 600 English bird or butterfly monitoring sites over three decades and tested how the surrounding land has influenced these changes. We partitioned community changes into warm‐ and cold‐associated assemblages and found that English bird communities have *not* reorganized successfully in response to climate change. CTI increases for birds are primarily attributable to the loss of cold‐associated species, whilst for butterflies, warm‐associated species have tended to increase. Importantly, the area of intensively managed land use around monitoring sites appears to influence these community changes, with large extents of intensively managed land limiting ‘adaptive’ community reorganization in response to climate change. Specifically, high‐intensity land use appears to exacerbate declines in cold‐adapted bird and butterfly species, and prevent increases in warm‐associated birds. This has broad implications for managing landscapes to promote climate change adaptation.

## Graphical abstract

This study investigates how bird and butterfly communities have changed during three decades of climate warming. We present the first evidence to show that community changes appear to be constrained by high‐intensity land use. This has broad implications for managing landscapes to promote climate change adaptation.

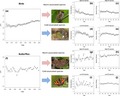

## Introduction

Climate change affects individual species differently, reflecting their contrasting environmental limits and requirements (Parmesan, 2006; Soberón, 2007), and may cause local extinctions and colonizations in any given location. This species turnover also has impacts on existing species through species interactions such as competition, predation, and mutualism (Cahill *et al*., 2013; Ockendon *et al*., 2014a). Therefore, as the climate changes, the balance of different species and thus the nature of ecological communities change (Devictor *et al*., 2008, 2012; Bellard *et al*., 2012).

There is also clear evidence that land‐use change can have profound impacts on biological communities (Lambin & Meyfroidt, 2011; Kampichler *et al*., 2012). Both land‐use and climate changes are expected to interact in their effects upon species (Oliver & Morecroft, 2014). For example, land‐use patterns affect the tendency of some species to shift their ranges in response to climate change (Warren *et al*., 2001) and can influence population (Newson *et al*., 2014; Oliver *et al*., 2015) and community (De Palma *et al*., 2016) responses to extreme climate events. However, little is known about the extent to which land‐use patterns affect the longer‐term capacity of biological communities to adapt in response to climate change.

Changes in community structure attributable to climate change have been previously captured through indicators such as the ‘community temperature index’ (CTI; Devictor *et al*., 2008, 2012). The CTI reflects changes in the balance of warm‐ and cold‐associated species in a given location. Species are first differentiated on the basis of the long‐term average temperature across their European range (the ‘species temperature index’; STI), reflecting their association with temperature. These STI scores are then weighted by species’ abundances to give a CTI score for any given location, expressed in degree Celsius. CTI scores of bird and butterfly assemblages across several European countries have been shown to increase over time, consistent with an expected response to a warming climate (Devictor *et al*., 2008, 2012). Therefore, the CTI has been viewed as reflecting the tendency of biological communities to ‘track’ climate change (Devictor *et al*., 2008; Settele *et al*., 2014; Gaüzère *et al*., 2016). The CTI metric has consequently been adopted as an indicator of climate change impact by the pan‐European framework supporting the Convention on Biological Diversity (Devictor *et al*., 2012).

**Figure 1:**
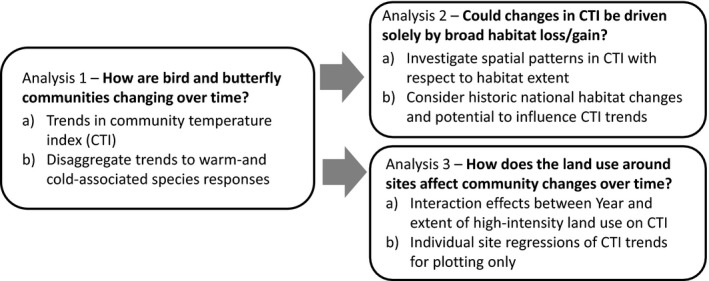
Schematic of the analytical workflow addressing three key questions regarding bird and butterfly community changes.

Indicators, by their nature, necessitate aggregation of complexity into simple metrics. These are useful for summarizing overall changes in communities, but it is important to recognize that they may mask contrasting trends in different components of the community. Here, we investigate changes in the species assemblages which underpin overall changes to community structure, to assess whether current interpretations of the CTI indicator are appropriate. We also investigate how land‐use patterns may interact with species assemblages to mediate responses. We use long‐term monitoring data from the UK Butterfly Monitoring Scheme (UKBMS; Pollard & Yates, 1993; Rothery & Roy, 2001) and the Common Birds Census (CBC; Marchant *et al*., 1990). These schemes represent an excellent source of data for this analysis as each site is sampled extensively by multiple visits throughout the year (UKBMS up to 26 visits and CBC sites up to 10 visits), and sampling is replicated across land‐use gradients, with sites being monitored over many years. Our analysis comprises three sequential components (Fig. 1).

First, we assess changes in CTI in England over time, as in a well‐cited study (Devictor *et al*., 2012). We then disaggregate the data to consider different abundance responses of cold‐ and warm‐associated species, allowing us to better understand the mechanisms underlying changes in CTI.

Second, we assess the extent to which changes in the CTI may be an artefact of other drivers such as habitat loss and degradation. Trends in individual species and the subsequent composition of biological communities are the outcome of multiple drivers of change (Brook *et al*., 2008; Burns *et al*., 2016), and these drivers could alter communities in ways that may obscure or outweigh climate change signals (Oliver *et al*., 2012). It has been suggested that changes in CTI may be driven by changes in habitat extent, due to correlations between species’ STI and habitat associations (Clavero *et al*., 2011; Barnagaud *et al*., 2012; Kampichler *et al*., 2012; Nieto‐Sánchez *et al*., 2015). Therefore, here we assess spatial patterns in the CTI of bird and butterfly communities with respect to land use around monitoring sites. We use these spatial relationships (where they exist) to ascertain whether land‐use change in England could be responsible for the observed changes in CTI.

Finally, we adopt a temporally explicit perspective and assess the degree to which community changes over time are mediated by the land use around monitoring sites. Climate change and land use are well known to interact in their impact on communities (Forister *et al*., 2010; Stefanescu *et al*., 2011; Eglington & Pearce‐Higgins, 2012; Oliver & Morecroft, 2014; Oliver *et al*., 2015). We hypothesize that large extents of intensively managed land may limit immigration of warm‐associated species (e.g. through reduced probability of colonization because intensively managed land is a barrier to dispersal; Dolman *et al*., 2007; Dover & Settele, 2009) and reduce the resilience of cold‐associated species (e.g. through reduced resource and microclimate availability; Oliver *et al*., 2010, 2013, 2015). We discuss the implications of our results both for the interpretation of the indicators of climate change such as CTI and for informing landscape management for climate change adaptation.

## Materials and methods

### Data sources

Butterfly data were available for 1976–2009 and bird data for 1964–2000. To maximize comparability in habitat composition between both schemes for our analysis (Fig. S1), we compared UKBMS sites, which are self‐selected and biased towards seminatural habitat (i.e. away from intensive agricultural and urban areas), with CBC sites centred on woodland. This restricted analyses to 454 and 159 English butterfly and bird monitoring sites, respectively. For consistency throughout, we present results for these sites with complete land‐cover data available, but CTI trends are qualitatively similar for a UK‐wide analysis (i.e. an additional 22 Scottish and 17 Welsh sites; results not shown). To assess bird and butterfly community composition, we used annual indices of abundance for 114 species (Table S1) and 63 species (Table S2), respectively, at CBC and UKBMS sites. For birds, these indices are the total number of individual territories in (c. 20 ha) survey areas calculated from 8 to 10 visits over the year by trained observers, recording bird presences based on sight and sound. Bird territories are mapped by British Trust for Ornithology experts using rigorous guidelines, to ensure consistency between estimates across sites and years (Marchant *et al*., 1990). Bird observers maintain the same sites over many years, which minimizes detectability biases affecting population trends (Marchant *et al*., 1990). Even if observers do change, this has very little effect on the results of territory analysis due to the extensive training of new observers (O'connor & Marchant, 1981). The sample of plots from which CBC results are drawn changed little in composition or character over the years (Marchant *et al*., 1990). For butterflies on UKBMS sites, our abundance indices are estimates of the total number of adult individuals calculated along 5‐m‐wide belt transects ranging from c. 2 to 5 km in length and from 20 to 26 visits throughout the flight period (Rothery & Roy, 2001). These have been shown to be good relative estimates of population size based on mark–release–recapture comparisons (Pollard & Yates, 1993). Detectability of butterflies can vary between sites, but variation in detectability is small compared with the variation in true abundance, such that population density estimates from the Pollard Walk transect method are highly correlated with those derived from distance sampling (Isaac *et al*., 2011). In addition, with little land‐use change over the study period (see Results), particularly on these UKBMS monitoring sites, detectability within sites should remain reasonably consistent, which is the important factor for these analyses of temporal trends in community structure. For both species recording schemes, we only used data for years in which at least 10 species were present at a site and for which abundance indices could be calculated for >75% of the species, so that our community metrics were reliable representations of actual species assemblages. Analyses were restricted to sites with more than five years’ data (*n *= 454 butterfly sites, *n *= 159 bird sites; Fig. S2), to allow robust assessment of community change over time.

To assess land use, we used remotely sensed 25‐m resolution CEH Land Cover Map 2000 (Fuller *et al*., 2002) and Natural England field habitat surveys http://www.gis.naturalengland.org.uk/pubs/gis/GIS_register.asp; accessed 1.3.2010), with land‐use information collated at radii of 0.5, 2, 5, and 10 km around monitoring site centroids (Fig. S1). We selected land‐cover maps which coincided approximately with the end of the monitoring periods, because we were interested in comparison of land cover among sites rather than change over time, of which there has been relatively little in the study period (see Results and Fig. S1). We assess ‘high‐intensity’ land use in terms of the area of various combinations of arable, urban, and improved grassland biotopes. These land uses are managed intensively and heavily modified from natural ecosystems in terms of their biophysical structure as well as water and nutrient cycling and other chemical inputs. Obviously, land‐use intensity can be further quantified within these human‐modified land‐use types (e.g. loads of fertilizer, herbicides, and pesticides applied to farmland), but such detailed management information is often lacking. Also, in comparison with other more natural land‐use types, such as calcareous grassland, broad management differences are implicit in our categorization of land cover; that is, management differences correlate strongly with the changes in the biophysical structure of land related to agricultural intensification, and thus, land‐use/land‐cover metrics encompass a large majority of the variations that affect species (Donald *et al*., 2001, 2006; Benton *et al*., 2002; Piha *et al*., 2007; Firbank *et al*., 2008; Ekroos *et al*., 2010; Oliver *et al*., 2010). Of our three high‐intensity land‐use types, the latter biotope category of intensive grassland is less well delineated by satellite remote sensing, and the two species groups may respond differently to each other. To account for this uncertainty, we fitted each combination of high‐intensity land use to the species data, using an information theoretic approach to identify the most appropriate metric and spatial scale for birds and butterflies. The metrics tested were the following combinations shown in Table 1. We also repeated analyses with all land‐cover combinations including sea in this grouping of potentially ‘hostile’ habitats. Climate data were obtained from the UK Met Office UKCP09 (2010) 5 km interpolated gridded monthly data set, and mean annual temperatures were calculated for each monitoring site and year.

**Table 1:** Combinations of land‐use categories used to define high‐intensity land use. Analyses were also repeated with all combinations below including sea as an additional potentially ‘hostile’ habitat. All combinations were assessed at four spatial scales of 0.5, 2, 5, and 10 km radius around monitoring site centroids. Results of statistical models testing the goodness of fit of these different characterizations for predicting changes in bird and butterfly species assemblages are shown in Tables S9 and S10

Combination	High‐intensity land use categorization	Abbreviation
1	Area of arable/horticultural land cover (A) from CEH Land Cover Map 2000 (LCM 2000)	A
2a	Arable area, A, from above plus improved grassland defined by the total grassland area from LCM2000 minus the area of calcareous grassland from field survey (IG1)	A + IG1
2b	Arable area, A, from above from above plus improved grassland defined by total grassland area from LCM2000 minus the area of calcareous grassland and lowland meadows from field survey (IG2)	A + IG2
3	Arable area, A, from above plus area of urban/suburban land use from LCM 2000	A + U
4a	Arable area, A, and urban area, U, from above, plus improved grassland defined as IG 1 above	A + U + IG1
4b	Arable area, A, and urban area, U, from above, plus improved grassland defined as IG 2 above	A + U + IG2

### Analysis of community trends over time

For all the species recorded on the monitoring sites, species temperature indices (STIs) were obtained from V. Devictor (i.e. as used in the paper Devictor *et al*., 2012), who based them on the average temperature occupied across their European range. Butterfly STI scores are now also openly available from Schweiger *et al*. (2014). Following the method in Devictor *et al*. (2008, 2012)), we calculated a ‘community temperature index’ (CTI) as the average STI of all bird or butterfly species present weighted by their relative abundance. Linear mixed‐effects models were used to test for temporal trends in CTI using *lme4* (Bates *et al*., 2013) in the program R (R Core Team, 2013). Year was fitted as a continuous fixed‐effect explanatory variable with random intercept terms for *Site* and *Year* (categorical variable), to account for the nonindependence of data within sites across years and across sites within years. A random slope term for the effect of *Year* at each site was also included because model comparison using AIC suggested that the temporal trend in CTI varied among sites (Appendix S1). Model diagnostic plots were checked to confirm residuals did not show patterning with respect to fitted values. Spatial autocorrelation was detected by extracting *Year* slopes for each site and plotting spline correlograms using the *ncf* package (Bjørnstad, 2009). To account for this, the 50‐km GB grid that each site occurred within was also included as a random intercept, effectively removing all spatial autocorrelation. Significance of the overall Year effect (i.e. for the direction and magnitude of the temporal trend in CTI) was assessed using a likelihood ratio test.

Next, within each group, birds or butterflies, we ranked species by their STI score and designated them based on quartiles (based on the median and interquartiles) into ‘low‐’, ‘medium–low‐’, ‘medium–high‐’, or ‘high‐’ STI species, indicating their association with either colder or warmer locations, respectively (Tables S3–S6). For each monitoring site and for each year, we calculated the total abundance and species richness in each of these four STI assemblages. Generalized linear mixed‐effects models were used to test for temporal trends in either total abundance or species richness in each of the four assemblages, with a Poisson error structure specified. Fixed and random effect structures were the same as for the analysis of the CTI response variable. We repeated all analyses for birds and butterflies using the same time period for both data sets (1976–2000) to assess whether there were qualitative differences in the results. We also repeated analyses excluding rare bird and butterfly species that may have higher sampling errors (e.g. a number of aquatic birds; Table S1; and rare butterflies: Table S2).

Finally, we also analysed abundance trends in each individual species to determine their contribution to the total abundance trend of low‐ or high‐STI species assemblages. For each species, we fitted a generalized linear mixed‐effects model with annual abundance as the response variable, Year (a continuous variable) as a fixed effect, and with a random intercept term for each *Site* and a Poisson error structure. The z value of this relationship (i.e. the change in number of individuals per year, weighted by standard error of this trend) approximately reflects a species contribution to the STI assemblage abundance trend.

### Analysis of spatial patterns in CTI with respect to local land use

We assessed whether the CTI of bird and butterfly communities varied spatially in relation to the composition of land cover on monitoring sites (defined using the smallest landscape radius of 0.5 km from monitoring route centroid). We fitted linear mixed‐effects models with CTI as the response variable and the extent of five land‐use types (% cover in the 0.5 km radius) as continuous fixed‐effect explanatory variables. Site northing and easting were also included as fixed effects, and *Site*,* Year,* and *50‐km GB grid* (all categorical variables) were fitted as random intercepts to account for repeated measures and variation in spatiotemporal CTI caused by other factors such as climate. Land‐use types investigated were the three high‐intensity land uses defined earlier (arable, improved grassland, urban/suburban) and two seminatural habitat types: broadleaved woodland and low‐intensity grassland, chosen because they are quite widespread seminatural habitat types. We repeated analyses using two alternative ways of defining improved and low‐intensity grassland (see Data sources). Where significant associations were found, we used the estimated coefficients to calculate what extent of land‐use change over time would be needed to fully explain the average national observed changes in CTI over time.

### Analysis of how land use mediates community changes over time

We next investigated how temporal trends in cold and warm species assemblages can be mediated by the extent of high‐intensity land use. For simplicity, we focussed here on changes in total abundance for the two species assemblages representing the most cold‐associated (i.e. low‐STI) or warm‐associated (i.e. high‐STI) species. We extended the generalized linear mixed‐effects statistical models from the first analysis (analysis of community trends over time*)* by adding interaction effects between Year and extent of high‐intensity land use. We also included interactions between Year and mean annual temperature to account for that fact that the local climate of a site may affect community changes. Hence, our response variable was the total abundance of low‐ or high‐STI species, and the fixed effects were Year (a continuous variable), total area of high‐intensity land use, local mean annual temperature, an interaction term between Year and total area of high‐intensity land use, and an interaction term between Year and local mean annual temperature. As before, random intercept terms were included for *Site*,* Year* and *50‐km GB Grid* (categorical variables), to account for the nonindependence of data within sites across years and across sites within years. We repeated these statistical models for each of the alternative definitions of high‐intensity land use (see Data sources) assessed at four spatial scales of 0.5, 2, 5, and 10 km radius around monitoring sites. We assessed model fit using the AIC criterion, allowing species data to inform on the most relevant categorization of high‐intensity land use and the appropriate spatial scale for each group. We also calculated conditional *R* ^2^ for each model in line with the method presented by Nakagawa and Schielzeth (Nakagawa & Schielzeth, 2013) and implemented in the MuMIn R package (Barton, 2015).

The statistical model fitted to all monitoring site data which included an interaction term between Year and total area of high‐intensity land use was the most rigorous approach to test our hypotheses; however, to illustrate our results, we fitted individual statistical models for each monitoring site to characterize temporal trends in the total abundance of low‐ and high‐STI species. We fitted a separate generalized linear model for each site, with the total abundance of low‐ or high‐STI species as the response and Year as the explanatory variable. A quasipoisson error structure was specified to account for overdispersion. We then plotted all the regression coefficients for these site‐specific temporal trends against the area of high‐intensity land use around each site. For this procedure, we omitted sites with fewer than six years’ data because, analysed individually in this manner, these sites are unlikely to provide reliable trend estimates.

## Results

### Bird and butterfly community trends over time

**Figure 2:**
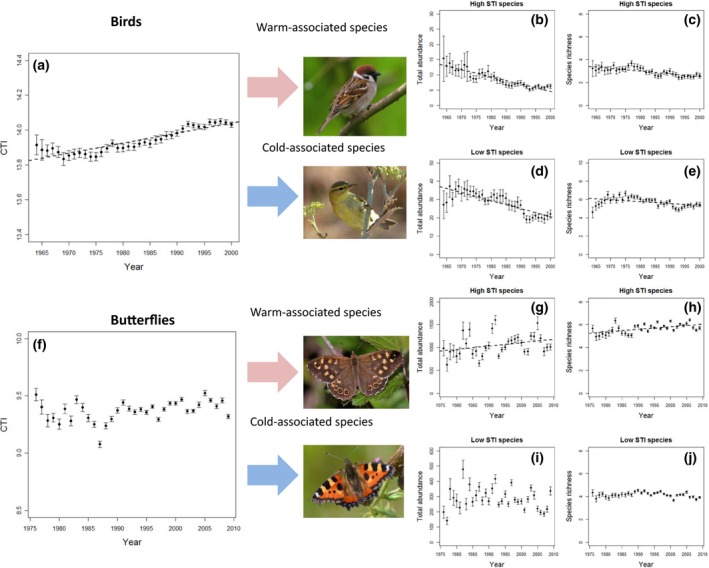
Changes in the balance of warm‐ and cold‐associated bird and butterfly species over time in England. Overall community change is reflected as a change in community temperature index (CTI, panels a and f), but can also be partitioned into changes in cold‐associated (low‐STI) or warm‐associated (high‐STI) species (panels b–e for birds and g‐j for butterflies). Plots are for mean values with error bars representing the spatial variation in community composition across all sites in any given year. Dashed lines indicate significant relationships (at *P* < 0.05).

We found that between 1964 and 2000 the CTI of English birds increased (CTI‐year coefficient = 0.0046 ± 0.0007, Χ^2^ = 489.8, *P *= <0.001; Fig. 2a). This is consistent, but more rapid, than equivalent changes in CTI observed at a European scale (Devictor *et al*., 2012). Importantly, disaggregation of bird communities into the most warm‐ and cold‐associated species (Tables S3–S4) showed that this change in CTI has been driven by a more rapid decline in the total abundance and species richness of low‐STI species, than that of high‐STI species, which also actually declined (Fig. 2). These assemblage declines were caused by a combination of both changes in the abundance of individual species (Tables S3 and S4) and the extinction of species from individual sites (Table 2; Figs 2 and S3). Warm‐associated birds in the third quartile of STI rankings (‘medium–high‐’ STI species) increased in abundance, albeit at a slower rate (by half) than the decline in low‐STI species (Table 1). Therefore, it appears that the observed increases in bird CTI have been driven primarily by the loss of cold‐associated species.

**Table 2:** Trends over time in the total abundance and species richness of bird and butterfly assemblages. Assemblages are defined through species ranked into quartiles by their species temperature indices (STI) from 1 (low STI) to 4 (high STI). Significant trends (at *P* < 0.05) are highlighted in bold

Group	Assemblage (STI quartile)	Total abundance trend				Species richness trend			
		Coefficient	SE	*z*	*P*	Coefficient	SE	*z*	*P*
Birds	High STI	−0.013	0.006	−2.23	**0.026**	−0.009	0.002	−5.09	**<0.001**
Birds	Medium–high STI	0.010	0.003	3.11	**0.002**	0.004	0.001	4.35	**<0.001**
Birds	Medium–low STI	0.006	0.004	1.69	0.091	−0.004	0.001	−4.42	**<0.001**
Birds	Low STI	−0.025	0.002	−11.27	**<0.001**	−0.007	0.001	−5.34	**<0.001**
Butterflies	High STI	0.260	0.042	6.14	**<0.001**	0.003	0.001	2.39	**0.017**
Butterflies	Medium–high STI	−0.004	0.007	−0.61	0.544	0.003	0.002	1.61	0.108
Butterflies	Medium–low STI	−0.006	0.004	−1.70	0.089	−0.001	0.001	−0.64	0.520
Butterflies	Low STI	−0.003	0.007	−0.49	0.621	−0.002	0.001	−1.83	0.068

Between 1976 and 2009, the CTI of English butterflies showed no significant change (CTI‐year coefficient = −0.0008 ± 0.0014, Χ^2^ = 0.30, *P* = 0.58; Fig. 2f). This is in contrast to Devictor *et al*. (2012) who found a significant increase and arises because in our case it was more appropriate to fit a statistical model with a random intercept for *Year*, in the light of the large interannual variability in CTI (e.g. Fig. 2f). Analysis of the data without this random effect and using all UK sites resulted in a significant increasing CTI trend (Appendix S1) as found by Devictor *et al*. (2012). Disaggregating the CTI of butterfly communities into warm‐ and cold‐associated species (Tables S5–S6) showed that, in contrast to birds, high‐STI butterflies have increased in total abundance and species richness, with no significant change in colder‐associated (low‐, medium–low‐, medium–high‐STI) species assemblages (Fig. 2; Table 2).

Repeating our analyses for birds and butterflies using same time period for both data sets (1976–2000), we found qualitatively similar results as for the full data set analysis (bird CTI‐year coefficient = 0.006 ± 0.0007, Χ^2^ = 51.4, *P* < 0.001; butterfly CTI‐year coefficient = −0.0027 ± 0.0025, Χ^2^ = 1.17, *P* = 0.28). Repeating our analyses excluding rare bird and butterfly species that may have higher sampling errors (e.g. a number of aquatic birds; Table S1; and rare butterflies: Table S2), the results were generally similar, except that the estimated rate of decline in high‐STI bird species over time was greater (Table S7). Again, the overall increase in bird CTI was due to the combined effects of a decline in low‐STI species and increase in ‘medium–high‐’ (i.e. upper third quartile) STI species, outweighing the high‐STI species declines. The trends for individual species that contribute to overall changes in the total abundance of STI assemblages can be found in Tables S3–S6.

### Spatial patterns in CTI with respect to land use

In testing whether the CTI of bird and butterfly communities varied spatially in relation to the composition of land cover around monitoring sites, we found that bird CTIs were higher on sites with more broadleaved woodland and urban areas, whilst butterfly CTIs were lower in woodland and grassland areas (Table S8). The slopes of these CTI–habitat area relationships suggest that for bird CTI changes during 1964–2009 (0.166 CTI units) to be solely driven by habitat would need a minimum increase in woodland cover from zero to 81% or in urban cover from zero to 85%, or some combination thereof. Current woodland and urban cover around sites already stand at 25% and 14%, respectively, and national net increases during 1990–2007 (where data are available) were 0.7% and 0.3%, respectively (Fig. S2).

### How land use and climate mediate community changes over time

We tested the effects of extent of high‐intensity land use and local climate on temporal trends in total abundance of cold (low‐STI)‐ and warm (high‐STI)‐butterfly and bird assemblages. Here, results are presented for the spatial scale and high‐intensity land‐use categorization which had the best ability to predict community changes. For high‐STI birds, this was arable land use, whilst for low‐STI birds it was arable and urban, both at 2 km radius. For low‐ and high‐STI butterflies, it was arable, urban, and improved grassland, both at 0.5 km radius. The goodness of fit of alternative models is detailed in Tables S9–S10, but results were qualitatively similar whichever method or spatial scale was used, and whether or not sea was included as a hostile habitat (Table S11). In the best‐fitting models, the conditional *R* ^2^ values were 0.90, 0.77 for low‐ and high‐STI birds, respectively, and 0.84 for both low‐ and high‐STI butterflies. The marginal *R* ^2^ values were 0.05, 0.06 for low‐ and high‐STI birds, respectively, and 0.03 for both low‐ and high‐STI butterflies. Thus, the majority of variation in abundance in both birds and butterflies is explained by the random effects in our models, reflecting the large interannual variation in abundance around long‐term trends (Fig. 2), plus the strong spatial patterns in abundance between sites and 50‐km regions.

**Figure 3:**
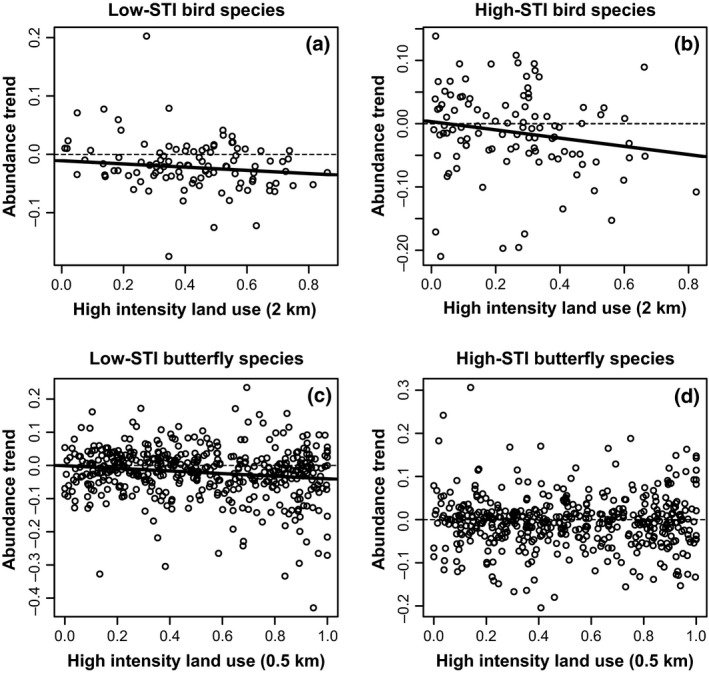
Associations between area of high‐intensity land use and change in abundance of low‐STI or high‐STI bird and butterfly species in England. Plotted are coefficients from regressions of abundance over time for each monitoring site with at least six years’ data. In the statistical analysis, however, all sites are analysed in a unified mixed model accounting for error in these temporal relationships, differences in site‐level intercepts, and spatial autocorrelation. Dashed lines on panels a–c indicate significant relationships.

For butterfly populations, we found that extent of high‐intensity land use was associated with more rapid declines in low‐STI species, but had no significant effect on high‐STI butterflies (Table 3; Fig. 3). In contrast, for birds, extent of high‐intensity land use was associated with declines in both low‐ and high‐STI bird species. With regard to mean annual temperature, for butterflies, we found a pattern whereby sites that were warmer over the recording period suffered more rapid decline in low‐STI species and were more likely to increase in high‐STI species, but this was not apparent for birds (Table 2). Results were qualitatively similar with rare species excluded from analyses (Table S12)

**Table 3:** Interaction effects between area of high‐intensity land use and year, and mean annual temperature and year, on the total abundance of low‐ or high‐STI bird and butterfly species. Interaction effects are demonstrated by plotting abundance trends over time vs. area of high‐intensity land use (Fig. 2). Significant interactions (at *P* < 0.05) are highlighted in bold

Species group	Response variable	Year: area high‐intensity land use coefficient	SE	*z*	*P*	Year: annual temperature coefficient	SE	*z*	*P*
Birds	Low‐STI species total abundance	−0.54	0.19	−2.82	**<0.001**	0.06	0.03	1.70	0.09
Birds	High‐STI species total abundance	−0.94	0.26	−3.60	**<0.001**	−0.03	0.04	−0.72	0.47
Butterflies	Low‐STI species total abundance	−0.28	0.09	−3.10	**0.002**	−0.15	0.01	−14.96	**<0.001**
Butterflies	High‐STI species total abundance	−0.0002	0.08	0.00	0.998	0.16	0.01	28.56	**<0.001**

## Discussion

### Bird and butterfly community trends over time

Our results demonstrate that English bird and butterfly communities show qualitatively different trends in their cold‐ and warm‐associated assemblages. This is despite similar directions of trend being found in an aggregate indicator of climate change impacts, the CTI (Devictor *et al*., 2012; also see Appendix S1). Butterfly communities appear to be responding adaptively to climate change through increases in warm‐associated species and relatively fewer declines across cold‐associated species. In contrast, birds have suffered declines in cold‐associated species but also declines in the most warm‐associated (high‐STI) species. This contrasts with results for two other countries, Sweden and North America, where both increases in warm‐associated species and declines in cold‐associated species were found to be responsible for the overall increases in CTI (Lindström *et al*., 2013; Princé & Zuckerberg, 2015; Tayleur *et al*., 2016).

The declines in English high‐STI bird species are potentially a result of habitat degradation (Thaxter *et al*., 2010; Eglington & Pearce‐Higgins, 2012). High‐STI birds suffered declines in both abundance and species richness (Fig. 2b and c). Although the coefficient for the rate of high‐STI bird abundance decline was roughly half that of low‐STI birds, the proportional loss is actually greater as initial starting abundances were lower, and when excluding rare species, the decline in abundance has been roughly equivalent between the two groups. Table S3 shows that within the most influential species (with high *t* values) affecting the total abundance trend of high‐STI assemblage, there are two main species types: species that are essentially ubiquitous within the range measured by the CBC and species that have expanded their range (Balmer *et al*., 2013). The former group mainly comprises widespread farmland species that declined over the time period due to factors linked to agricultural intensification (e.g. *Carduelis cannabina, Carduelis carduelis, Emberiza calandra, Passer montanus, Perdix perdix, Streptopelia turtur*; Chamberlain *et al*., 2000) that have been only partly balanced out by species that have shown population increases (e.g. *Corvus monedula, Fulica atra, Streptopelia decaocto, Strix aluco;* Baillie *et al*., 2014). The latter group includes species that have expanded their ranges northwards (e.g. *Alcedo atthis, Saxicola rubicola, Sitta europaea, Tachybaptus ruficollis*), but occur at relatively low population densities (Balmer *et al*., 2013). In addition to agricultural intensification in England, migratory species that overwinter elsewhere will be affected by habitat and weather conditions in their overwintering grounds (Finch *et al*., 2014; Ockendon *et al*., 2014b), and particularly, during the study period, arid‐zone African migrants declined during the 1970s due to Sahel drought, whilst humid‐zone African migrants have declined more recently (Thaxter *et al*., 2010).

The steeper declines of the low‐STI bird species appear to be related to climate‐related contraction from the south of their ranges of a number of more northerly distributed species, such as *Anthus pratensis, Carduelis cabaret, Phylloscopus trochilus, Poecile montana*. However, there are also examples of such species showing declines that are most likely due to land‐use change, as found for the high‐STI species, such as *Prunella modularis* and *Pyrrhula pyrrhula* (Balmer *et al*., 2013; Baillie *et al*., 2014).

For butterflies, the overall increases in high‐STI species are likely to have been driven by increases in abundance of widespread warm‐associated species such as *Pararge aegeria,* with additional contributions from habitat specialist species such as *Polyommatus bellargus* and the migrant *Colias croceus*. There are also general increases in the species richness of high‐STI species on monitoring sites over recent decades (Fig. 2). These changes are all compatible with climate warming, although improved management of protected areas may have provided additional benefits for some specialist species (Fox *et al*., 2006; Thomas *et al*., 2012b). However, it should be noted that some high‐STI species, such as *Lasiommata megera,* are exceptions to this trend and are suffering unexplained declines in occurrence and local abundance in England and wider Europe (Klop *et al*., 2015; Van Dyck *et al*., 2015).

For low‐STI butterflies, there was an overall lack of change in abundance or species richness, possibly because butterflies are thermophilic insects and there are relatively few species in high‐latitude countries such as England for which the climate is yet too warm (Settele *et al*., 2008; Oliver *et al*., 2014). However, that may well change in the future with climate warming, in particular with an increased frequency of extreme events such as droughts (Oliver *et al*., 2015).

### Spatial patterns in CTI with respect to land use

An issue previously raised with aggregate climate indicators such as CTI is that, if there are correlations between species’ STI and habitat associations, then changes in CTI may be driven by changes in habitat extent (Clavero *et al*., 2011; Barnagaud *et al*., 2012; Kampichler *et al*., 2012; Nieto‐Sánchez *et al*., 2015). If this is the case, then one would expect there to be strong spatial patterns in CTI with respect to the habitat composition of monitoring sites. We tested for such relationships but only found limited evidence. There were associations between CTI scores and the extent of some habitat types (woodland and urban for birds, and woodland and grassland for butterflies). The positive association of bird CTI and woodland extent was opposite to that hypothesized for Central Europe (i.e. where lower‐STI species were more likely to be woodland‐associated species, meaning an increase in woodland should decrease CTI scores; Clavero *et al*., 2011). The change in the CTI along spatial gradients of habitat extent that we observed was relatively small. For example, to explain fully the national average increase in bird CTI during 1964–2009 would require increases in woodland or urban extent of up to approximately 80% cover. Recent rates of land‐cover change in England have been much lower (e.g. there has been <1% total cover change in these habitats over 18 years between land‐cover maps of 1990 and 2007). Therefore, changes in *habitat extents* on monitoring sites themselves are unlikely to be the main driver of community changes observed in our analyses (note, this does not preclude changes in habitat *quality* strongly affecting community composition).

### Interactions between extent of high‐intensity land use and local climate

A more complete understanding of community changes should help inform upon ways to reverse detrimental trends as well as simply documenting them. Land‐use and climate changes are well known to interact in their effects on species (Oliver & Morecroft, 2014). At the community level, we investigated the combined outcome of these species effects by considering how the area of high‐intensity‐use land cover mediates community changes over time. For butterfly populations, we found that extent of high‐intensity land use was associated with more rapid declines in low‐STI species (Table 3; Fig. 3). This supported our hypothesis that large extents of intensively managed land reduce the resilience of cold‐associated species, potentially mediated through a reduction in the availability and diversity of resources and suitable microclimates, and further exacerbated by reduced metapopulation size and increased risk of stochastic extinctions (Hanski & Ovaskainen, 2000; Oliver *et al*., 2010, 2013, 2015; Lawson *et al*., 2012; Gillingham *et al*., 2015). In contrast, the extent of high‐intensity land use had no significant effect on high‐STI butterflies (Table 3; Fig. 3), and thus, there was no support for our hypothesis that larger extents of high‐intensity land use limit immigration success and consequent population growth of warm‐associated butterflies. For birds, extent of high‐intensity land use was associated with declines in both low‐ and high‐STI bird species, perhaps indicating a strong main effect of habitat degradation rather than an interaction with species’ thermal tolerances. Overall, our results suggest that land use can influence adaptive community responses to climate change but that different species groups respond differently.

In terms of the most appropriate scale to target adaptation measures, our results suggest that bird communities will respond to landscape management at larger spatial scales (i.e. 2 km radius around monitoring sites), whereas butterfly communities will respond more to local land‐use changes (i.e. at a 0.5 km radius). Arable/horticultural and urban/suburban land cover both had negative effects on butterflies and cold‐associated (low‐STI) birds, whilst urban land cover was not included as a ‘hostile’ land use for high‐STI birds. For butterflies, intensive grassland was also included in the most appropriate grouping of ‘hostile’ land use, probably reflecting the lack of resources and even negative impacts of this land‐use type (Wallisdevries & Van Swaay, 2006).

With regard to how the local climate for each site affected changes in low and high STI in assemblages, for butterflies, we found a pattern whereby sites that were warmer over the recording period suffered more rapid decline in low‐STI species and were more likely to increase in high‐STI species (Table 2), further supporting climate, in addition to habitat quality, as important factor mediating these community changes. This was not apparent for birds perhaps indicating a greater influence of habitat degradation for these communities (Eglington & Pearce‐Higgins, 2012).

In summary, when assessing the effects of land‐use and climate changes on communities, it seems it is important to look beyond generic indicators such as CTI, which can mask qualitatively different patterns between species groups. Our results suggest that bird communities are reorganizing less successfully in response to climate change than butterflies, because increases in bird CTI are driven by greater loss of cold‐ than gain in warm‐associated species. Thus, the CTI actually can give false assurance for some communities ‘tracking’ climate change (Devictor *et al*., 2008; Gaüzère *et al*., 2016), as appears to be the case for birds in England. Many bird species (including high‐STI birds) have probably declined due to declines in habitat quality, although the relatively greater loss of low‐STI species than high‐STI species is supportive of an additional climate change element to these community changes. In contrast, for butterflies, high‐STI species have increased, with little overall changes to the cold‐associated components of communities. These patterns are mostly consistent with climate warming impacts, and our analysis supports the assertion that criticisms of the CTI indicator based on responsiveness to habitat change are unlikely to fully explain the national changes in CTI (Gaüzère *et al*., 2015). However, degradation of habitat quality may certainly contribute to community changes and can cause unexpected patterns in species assemblages (e.g. high‐STI bird declines) that are masked by the use of an aggregate indicator. In addition, we show that the extent of high‐intensity land use may also interact with climate change to limit autonomous community reorganization. Hostile land use appears to exacerbate declines in cold‐adapted species, with an additional effect of preventing increases in warm‐associated birds. These results potentially account for recent evidence that protected areas can enhance species’ persistence and colonization under climate warming (Thomas *et al*., 2012a; Gillingham *et al*., 2015; Oliver *et al*., 2015). Further research considering rates of community change in the light of climatic variability and whether interactions with land use are mediated by ecological traits (e.g. De Palma *et al*., 2016) and regional species pools would provide additional valuable insights into changing biological communities under global change drivers.

## Supporting information


**Figure S1.** Proportion of broad land use types around monitoring sites.
**Figure S2.** Duration of recording at each monitoring site.
**Figure S3.** Changes in total abundance and species richness of all bird and butterfly species over time.
**Table S1.** Full list of bird species found across the 159 Common Bird Census monitoring sites.
**Table S2.** Full list of butterfly species found across the 454 Butterfly Monitoring Scheme sites.
**Table S3.** Warm‐associated (high STI) bird species.
**Table S4.** Cold‐associated (low STI) bird species.
**Table S5.** Warm‐associated (high STI) butterfly species.
**Table S6.** Cold‐associated (low STI) butterfly species.
**Table S7.** Sensitivity analysis of trends over time in the total abundance and species richness of bird and butterfly assemblages excluding rare species.
**Tables S8.** Relationships between bird and butterfly CTI scores and habitat extent around monitoring sites.
**Table S9.** Comparison of model goodness of fit for different land cover characterisations.
**Table S10.** Comparison of model goodness of fit for different land cover characterisations including the area of sea as hostile habitat.
**Table S11.** Model results using alternative classification of high‐intensity land cover or at different spatial scales.
**Table S12.** Sensitivity analysis of interactions of bird and butterfly assemblages with land use and climate excluding rare species.
**Appendix S1.** Comparison of butterfly community temperature index (CTI) trends with Devictor *et al*.(2012).
**Table S13.** Models with varying random effect structures for assessing temporal trends in butterfly community temperature index (CTI) across the UK.
**Table S14.** Models assessing temporal trends in butterfly community temperature index (CTI) in three UK countries.
**Table S15.** Models with varying random effect structures for assessing temporal trends in butterfly community temperature index (CTI) in England.
