# Supplementary material for: Large extents of intensive land use limit community reorganization during climate warming
Source: Glob Chang Biol. 2017 Jan 10;23(6):2272–83. doi: 10.1111/gcb.13587 (PMC6849802; doi:10.1111/gcb.13587)
Supplement: Supplementary file 1 — Figure S1. Proportion of broad land use types around monitoring sites. Figure S2. Duration of recording at each monitoring site. Figure S3. Changes in total abundance and species richness of all bird and butterfly species over time. Table S1. Full list of bird species found across the 159 Common Bird Census monitoring sites. Table S2. Full list of butterfly species found across the 454 Butterfly Monitoring Scheme sites. Table S3. Warm‐associated (high STI) bird species. Table S4. Cold‐associated (low STI) bird species. Table S5. Warm‐associated (high STI) butterfly species. Table S6. Cold‐associated (low STI) butterfly species. Table S7. Sensitivity analysis of trends over time in the total abundance and species richness of bird and butterfly assemblages excluding rare species. Tables S8. Relationships between bird and butterfly CTI scores and habitat extent around monitoring sites. Table S9. Comparison of model goodness of fit for different land cover characterisations. Table S10. Comparison of model goodness of fit for different land cover characterisations including the area of sea as hostile habitat. Table S11. Model results using alternative classification of high‐intensity land cover or at different spatial scales. Table S12. Sensitivity analysis of interactions of bird and butterfly assemblages with land use and climate excluding rare species. Appendix S1. Comparison of butterfly community temperature index (CTI) trends with Devictor et al.(2012). Table S13. Models with varying random effect structures for assessing temporal trends in butterfly community temperature index (CTI) across the UK. Table S14. Models assessing temporal trends in butterfly community temperature index (CTI) in three UK countries. Table S15. Models with varying random effect structures for assessing temporal trends in butterfly community temperature index (CTI) in England. [file GCB-23-2272-s001.doc]

**Supporting Information**

**This Supporting Information contains:**

**Figure S1-** Proportion of broad land use types around monitoring sites

**Figure S2-** Duration of recording at each monitoring site

**Figure S3-** Changes in total abundance and species richness of all bird and butterfly species over time

**Table S1**- Full list of bird species found across the 159 Common Bird Census monitoring sites

**Table S2**- Full list of butterfly species found across the 454 Butterfly Monitoring Scheme sites

**Table S3**- Warm-associated (high STI) bird species

**Table S4**- Cold-associated (low STI) bird species

**Table S5-** Warm-associated (high STI) butterfly species

**Table S6-** Cold-associated (low STI) butterfly species

**Table S7**- Sensitivity analysis of trends over time in the total abundance and species richness of bird and butterfly assemblages excluding rare species

**Tables S8** Relationships between bird and butterfly CTI scores and habitat extent around monitoring sites

**Table S9-** Comparison of model goodness of fit for different land cover characterisations

**Table S10-** Comparison of model goodness of fit for different land cover characterisations including the area of sea as hostile habitat

**Table S11-** Model results using alternative classification of high intensity land cover or at different spatial scales

**Table S12-** Sensitivity analysis of interactions of bird and butterfly assemblages with land use and climate excluding rare species

**Appendix S1-** Comparison of butterfly community temperature index (CTI) trends with Devictor et al..

**Table S13-** Models with varying random effect structures for assessing temporal trends in butterfly community temperature index (CTI) across the UK

**Table S14-** Models assessing temporal trends in butterfly community temperature index (CTI) in three UK countries

**Table S15-** Models with varying random effect structures for assessing temporal trends in butterfly community temperature index (CTI) in England

**Figure S1**, Proportion of broad land-use types around a) butterfly and b) bird monitoring sites. Dark shaded land uses represent those categorised as high intensity for that group through the best fitting statistical models showing a significant impact on species assemblage change (see Tables S10-S12). The four bars for each land use represent areas assessed at 0.5, 2, 5 and 10km radii around monitoring sites (from left to right). Abbreviations are as follows: A- Arable cereals, arable horticulture, non-rotational horticulture; IG1 & IG2- Improved grassland from all grassland cover from remote sensing data minus lowland calcareous grassland from field surveys (IG1) or lowland calcareous grassland plus lowland meadows (IG2; *The asterisks indicate alternative definitions and hence proportions do not sum to unity); U- Urban or suburban landcover; Br- Bracken; BW- Broadleaved woodland; C- Coastal (comprised of littoral rock or sediment, saltmarsh, supra-littoral rock, supra-littoral sediment; CW- Coniferous woodland; F- Fen, marsh or swamp; H- Dwarf shrub heath; LIG1 & LIG2 – alternative definitions of low intensity grassland (lowland calcareous grassland or lowland calcareous grassland plus lowland meadows); R- rivers, inland water; S- Open sea or estuary. Land use metrics were derived from CEH Landcover Map 2000 to coincide approximately with the end of the monitoring periods. Most major English land use conversions occurred in the early half of the 20^th^ Century. National data available from 1990-2007 show that recent changes comprise increases in broadleaved woodland (6.7 to 7.4% total area), coniferous woodland (1.8 to 1.9%), urban/suburban (7.6-7.9%) and dwarf shrub heath (2.4 to 2.5%). In contrast, extent of arable and horticulture declined (33.2 to 30.4%) as did calcareous grassland (0.3 to 0.2%).


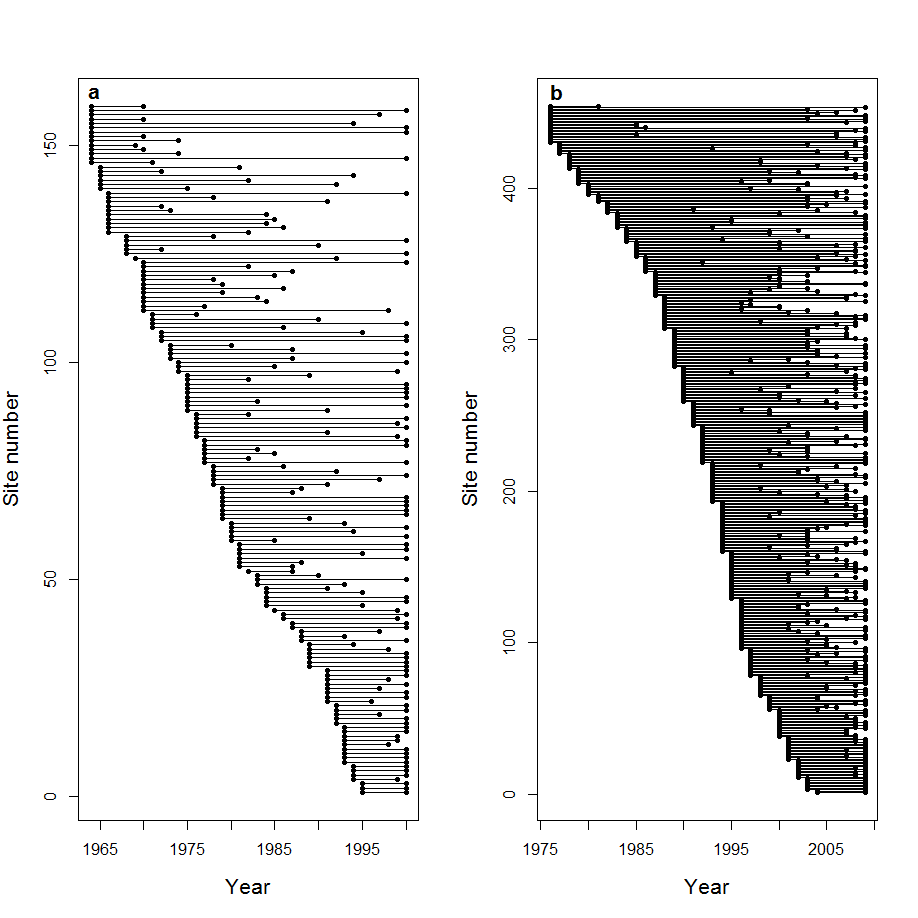


**Figure S2**, Duration of recording at each a) Common Bird Census (n = 159), and b) UK Butterfly Monitoring Scheme site (n = 454) in the analysis.


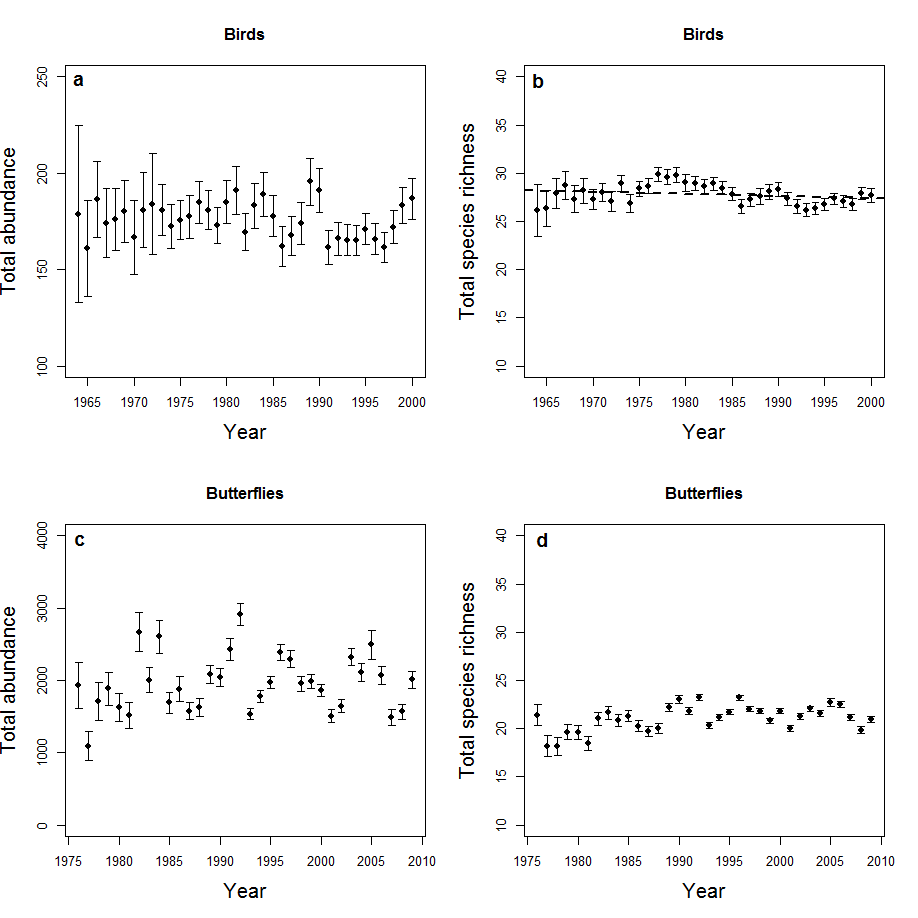


**Figure S3**, Changes in total abundance and species richness of all bird and butterfly species over time. Plots are for mean values with error bars representing the spatial variation in community composition across all sites in any given year. Dashed lines indicate significant relationships (at p < 0.05).

**Table S1- Full list of bird species found across the 159 Common Bird Census monitoring sites**. Less common species occurring on fewer than 10 sites (indicated by asterisks) and nocturnal species (†) were excluded in a sensitivity analysis, as these species may have higher sampling errors. *Passer domesticus* (‡) was also excluded as this species was not recorded consistently during the early years of the CBC scheme.

**Table S2- Full list of butterfly species found across the 454 Butterfly Monitoring Scheme sites.** Less common species occurring on fewer than 10 sites are indicated by asterisks and were excluded in a sensitivity analysis, as these species may have higher sampling errors.

**Table S3**- **Warm-associated (high STI) bird species**, identified as those with species temperature indices (STIs) in the top quartile of bird species found on the monitoring sites. Provided are statistics for individual abundance trends for each species occurring on more than one site (calculated from a mixed model of count versus year with a random intercept for each site) along with the total species count on all sites across all years. Species are ranked by t-value which approximately reflects their contribution to the overall high STI total abundance trend (i.e. the change in number of individuals of that species per year, weighted by standard error of this trend). Note that loss or gain of rare species contributes very little to the CTI index, which is weighted by abundance.

| **Latin name** | **Common name (English)** | **STI score** | **Abundance trend coefficient** | **se** | **t** | **p** | **Total counts 1964-2000** | **Number non-zero sites** |
| --- | --- | --- | --- | --- | --- | --- | --- | --- |
| *Passer montanus* | Tree Sparrow | 15.04 | -0.11 | 0.004 | -25.85 | <0.001 | 1651 | 45 |
| *Carduelis cannabina* | Linnet | 15.05 | -0.07 | 0.003 | -22.40 | <0.001 | 2633 | 73 |
| *Streptopelia turtur* | Turtle Dove | 15.72 | -0.05 | 0.003 | -15.69 | <0.001 | 2287 | 79 |
| *Luscinia megarhynchos* | Nightingale | 16.25 | -0.04 | 0.004 | -9.62 | <0.001 | 850 | 44 |
| *Perdix perdix* | Grey Partridge | 14.81 | -0.10 | 0.01 | -9.36 | <0.001 | 211 | 26 |
| *Sitta europaea* | Nuthatch | 14.75 | 0.02 | 0.003 | 8.33 | <0.001 | 3666 | 123 |
| *Coccothraustes coccothraustes* | Hawfinch | 14.97 | -0.10 | 0.02 | -6.55 | <0.001 | 128 | 23 |
| *Carduelis carduelis* | Goldfinch | 15.37 | -0.03 | 0.01 | -5.95 | <0.001 | 607 | 73 |
| *Emberiza calandra* | Corn Bunting | 16.04 | -0.17 | 0.03 | -5.05 | <0.001 | 35 | 7 |
| *Milvus milvus* | Red Kite | 15.34 | 0.27 | 0.06 | 4.79 | <0.001 | 25 | 2 |
| *Streptopelia decaocto* | Collared Dove | 14.79 | 0.02 | 0.005 | 4.71 | <0.001 | 1055 | 66 |
| *Rallus aquaticus* | Water Rail | 15.15 | -0.16 | 0.04 | -4.07 | <0.001 | 17 | 2 |
| *Caprimulgus europaeus* | Nightjar | 15.06 | -0.06 | 0.02 | -3.83 | <0.001 | 85 | 10 |
| *Saxicola rubicola* | Stonechat | 15.77 | -0.08 | 0.03 | -2.54 | 0.011 | 36 | 5 |
| *Fulica atra* | Coot | 14.87 | 0.02 | 0.01 | 2.49 | 0.013 | 392 | 17 |
| *Acrocephalus scirpaceus* | Reed Warbler | 15.06 | -0.03 | 0.01 | -2.47 | 0.014 | 222 | 9 |
| *Tachybaptus ruficollis* | Little Grebe | 15.26 | 0.04 | 0.02 | 2.20 | 0.028 | 80 | 11 |
| *Lullula arborea* | Woodlark | 15.43 | 0.05 | 0.03 | 2.00 | 0.046 | 42 | 6 |
| *Corvus monedula* | Jackdaw | 14.89 | 0.00 | 0.00 | 1.68 | 0.093 | 2597 | 84 |
| *Tyto alba* | Barn Owl | 15.62 | -0.05 | 0.04 | -1.20 | 0.231 | 6 | 5 |
| *Gallinula chloropus* | Moorhen | 15.25 | 0.00 | 0.00 | -0.95 | 0.340 | 1186 | 69 |
| *Regulus ignicapilla* | Firecrest | 15.07 | 0.01 | 0.02 | 0.82 | 0.412 | 95 | 7 |
| *Strix aluco* | Tawny Owl | 14.77 | 0.00 | 0.004 | 0.39 | 0.699 | 1076 | 130 |
| *Sylvia undata* | Dartford Warbler | 17.16 | -0.01 | 0.04 | -0.33 | 0.739 | 7 | 3 |
| *Alcedo atthis* | Kingfisher | 15.44 | 0.00 | 0.03 | 0.10 | 0.917 | 30 | 10 |
| *Oriolus oriolus* | Golden Oriole | 15.52 | - | - | - | - | 4 | 1 |
| *Falco peregrinus* | Peregrine | 14.95 | - | - | - | - | 1 | 1 |
| *Cettia cetti* | Cetti's Warbler | 17.35 | - | - | - | - | 1 | 1 |

**Table S4**- **Cold-associated (low STI) bird species**, identified as those with species temperature indices (STIs) in the bottom quartile of bird species found on the monitoring sites. Provided are statistics for individual abundance trends for each species occurring on more than one site (calculated from a mixed model of count versus year with a random intercept for each site) along with the total species count on all sites across all years. Species are ranked by t-value which approximately reflects their contribution to the overall high STI total abundance trend (i.e. the change in number of individuals of that species per year, weighted by standard error of this trend). Note that loss or gain of rare species contributes very little to the CTI index, which is weighted by abundance.

| **Latin name** | **Common name (English)** | **STI score** | **Abundance trend coefficient** | **se** | **t** | **p** | **Total counts 1964-2000** | **Number non-zero sites** |
| --- | --- | --- | --- | --- | --- | --- | --- | --- |
| *Prunella modularis* | Dunnock | 12.81 | -0.032 | 0.001 | -26.21 | <0.001 | 15814 | 153 |
| *Phylloscopus trochilus* | Willow Warbler | 12.45 | -0.023 | 0.001 | -23.10 | <0.001 | 23041 | 160 |
| *Carduelis cabaret* | Redpoll | 8.19 | -0.071 | 0.005 | -15.18 | <0.001 | 1333 | 63 |
| *Corvus corone/cornix* | Carrion/Hooded Crow | 13.11 | 0.031 | 0.003 | 11.26 | <0.001 | 3276 | 152 |
| *Anthus pratensis* | Meadow Pipit | 11.83 | -0.056 | 0.006 | -9.09 | <0.001 | 432 | 16 |
| *Pyrrhula pyrrhula* | Bullfinch | 12.66 | -0.018 | 0.002 | -8.35 | <0.001 | 4627 | 148 |
| *Anthus trivialis* | Tree Pipit | 13.25 | -0.029 | 0.004 | -7.13 | <0.001 | 1300 | 60 |
| *Regulus regulus* | Goldcrest | 12.69 | -0.015 | 0.002 | -6.75 | <0.001 | 5755 | 148 |
| *Poecile montana* | Willow Tit | 12.26 | -0.034 | 0.005 | -6.24 | <0.001 | 800 | 75 |
| *Aythya fuligula* | Tufted Duck | 12.07 | 0.063 | 0.014 | 4.64 | <0.001 | 132 | 11 |
| *Scolopax rusticola* | Woodcock | 12.59 | -0.030 | 0.007 | -4.34 | <0.001 | 426 | 62 |
| *Corvus corax* | Raven | 11.74 | 0.228 | 0.067 | 3.43 | 0.001 | 33 | 12 |
| *Chloris chloris* | Greenfinch | 12.60 | -0.008 | 0.002 | -3.37 | 0.001 | 3540 | 115 |
| *Carduelis spinus* | Siskin | 12.17 | 0.084 | 0.033 | 2.56 | 0.010 | 34 | 14 |
| *Ficedula hypoleuca* | Pied Flycatcher | 12.53 | 0.031 | 0.018 | 1.74 | 0.081 | 295 | 16 |
| *Numenius arquata* | Curlew | 11.91 | 0.030 | 0.019 | 1.61 | 0.106 | 55 | 8 |
| *Gallinago gallinago* | Snipe | 12.17 | -0.036 | 0.033 | -1.10 | 0.269 | 10 | 6 |
| *Mergus merganser* | Goosander | 9.97 | 0.048 | 0.048 | 1.00 | 0.319 | 8 | 2 |
| *Certhia familiaris* | Treecreeper | 13.07 | 0.002 | 0.003 | 0.64 | 0.519 | 3438 | 144 |
| *Actitis hypoleucos* | Common Sandpiper | 13.20 | -0.035 | 0.061 | -0.58 | 0.565 | 21 | 2 |
| *Loxia curvirostra* | Crossbill | 12.46 | 0.003 | 0.037 | 0.08 | 0.933 | 15 | 7 |
| *Cinclus cinclus* | Dipper | 12.85 | 0.001 | 0.033 | 0.03 | 0.980 | 37 | 5 |
| *Anas crecca* | Teal | 11.97 | - | - | - | - | 21 | 1 |
| *Anser anser* | Greylag Goose | 12.72 | - | - | - | - | 8 | 1 |
| *Bucephala clangula* | Goldeneye | 10.71 | - | - | - | - | 1 | 1 |
| *Haematopus ostralegus* | Oystercatcher | 12.11 | - | - | - | - | 9 | 1 |
| *Mergus serrator* | Red-breasted Merganser | 9.44 | - | - | - | - | 8 | 1 |
| *Tringa totanus* | Redshank | 13.00 | - | - | - | - | 2 | 1 |
| *Turdus torquatus* | Ring Ousel | 10.74 | - | - | - | - | 1 | 1 |

**Table S5- Warm-associated (high STI) butterfly species**, identified as those with species temperature indices (STIs) in the top quartile of butterfly species found on the monitoring sites. Provided are statistics for individual abundance trends for each species occurring on more than one site (calculated from a mixed model of count versus year with a random intercept for each site) along with the total species count on all sites across all years. Species are ranked by t-value which approximately reflects their contribution to the overall high STI total abundance trend (i.e. the change in number of individuals of that species per year, weighted by standard error of this trend). Note that loss or gain of rare species contributes very little to the CTI index, which is weighted by abundance.

| **Latin name** | **Common name (English)** | **STI score** | **Abundance trend coefficient** | **se** | **t** | **p** | **Total counts 1976-2009** | **Number non-zero sites** |
| --- | --- | --- | --- | --- | --- | --- | --- | --- |
| *Pararge aegeria* | Speckled wood | 9.71 | 0.0239 | 0.0002 | 100.71 | <0.001 | 535005 | 446 |
| *Lasiommata megara* | Wall brown | 10.39 | -0.0594 | 0.0006 | -94.01 | <0.001 | 62728 | 308 |
| *Pyronia tithonus* | Gatekeeper | 10.86 | -0.0140 | 0.0002 | -90.57 | <0.001 | 1281802 | 438 |
| *Polyommatus bellargus* | Adonis blue | 10.19 | 0.0303 | 0.0004 | 72.21 | <0.001 | 106038 | 70 |
| *Pieris rapae* | Small white | 9.63 | -0.0115 | 0.0002 | -47.49 | <0.001 | 472161 | 454 |
| *Thymelicus acteon* | Lulworth skipper | 11.31 | -0.0549 | 0.0013 | -42.30 | <0.001 | 18887 | 5 |
| *Colias croceus* | Clouded yellow | 10.69 | 0.0365 | 0.0016 | 22.64 | <0.001 | 11779 | 382 |
| *Melanargia galathea* | Marbled white | 9.71 | 0.0042 | 0.0002 | 16.92 | <0.001 | 497065 | 340 |
| *Melitaea cinxia* | Glanville fritillary | 9.60 | -0.0720 | 0.0083 | -8.69 | <0.001 | 401 | 4 |
| *Euphydryas aurinia* | Marsh fritillary | 9.53 | 0.0133 | 0.0015 | 8.58 | <0.001 | 13166 | 57 |
| *Neozephyrus quercus* | Purple hairstreak | 9.49 | 0.0106 | 0.0015 | 6.94 | <0.001 | 15395 | 242 |
| *Aricia agestis* | Brown argus | 10.16 | 0.0017 | 0.0006 | 3.10 | 0.002 | 89475 | 305 |
| *Maniola jurtina* | Meadow brown | 9.85 | 0.0002 | 0.0001 | 2.51 | 0.012 | 3433247 | 454 |
| *Nymphalis polychloros* | Large tortoiseshell | 9.68 | -0.0388 | 0.0486 | -0.80 | 0.425 | 19 | 9 |
| *Colias alfacariensis* | Berger's clouded Yellow | 9.94 | -0.0374 | 0.0593 | -0.63 | 0.528 | 15 | 4 |

**Table S6**- **Cold-associated (low STI) butterfly species**, identified as those with species temperature indices (STIs) in the bottom quartile of butterfly species found on the monitoring sites. Provided are statistics for individual abundance trends for each species occurring on more than one site (calculated from a mixed model of count versus year with a random intercept for each site) along with the total species count on all sites across all years. Species are ranked by t-value which approximately reflects their contribution to the overall high STI total abundance trend (i.e. the change in number of individuals of that species per year, weighted by standard error of this trend). Note that loss or gain of rare species contributes very little to the CTI index, which is weighted by abundance.

| **Latin name** | **Common name (English)** | **STI score** | **Abundance trend coefficient** | **se** | **t** | **p** | **Total counts 1976-2009** | **Number non-zero sites** |
| --- | --- | --- | --- | --- | --- | --- | --- | --- |
| *Aphantopus hyperantus* | Ringlet | 7.90 | 0.0224 | 0.0002 | 120.02 | <0.001 | 764103 | 393 |
| *Aglais urticae* | Small tortoiseshell | 7.87 | -0.0328 | 0.0003 | -102.39 | <0.001 | 282635 | 454 |
| *Argynnis aglaja* | Dark green fritillary | 7.79 | 0.0389 | 0.0008 | 47.68 | <0.001 | 41001 | 200 |
| *Aricia artaxerxes* | Northern brown argus | 6.45 | -0.0577 | 0.0015 | -38.24 | <0.001 | 16713 | 18 |
| *Boloria euphrosyne* | Pearl-bordered fritillary | 6.95 | -0.0423 | 0.0012 | -35.98 | <0.001 | 20979 | 80 |
| *Boloria selene* | Small pearl-bordered fritillary | 6.93 | -0.0384 | 0.0015 | -26.01 | <0.001 | 16285 | 79 |
| *Pyrgus malvae* | Grizzled skipper | 8.03 | -0.0171 | 0.0011 | -15.60 | <0.001 | 26044 | 203 |
| *Erebia aethiops* | Scotch argus | 8.10 | -0.0152 | 0.0012 | -13.12 | <0.001 | 29623 | 7 |
| *Pieris napi* | Green-veined white | 8.21 | -0.0030 | 0.0002 | -12.99 | <0.001 | 429571 | 453 |
| *Coenonympha tullia* | Large heath | 6.36 | 0.0609 | 0.0051 | 11.85 | <0.001 | 1975 | 4 |
| *Melitaea athalia* | Heath fritillary | 8.04 | -0.0214 | 0.0021 | -10.43 | <0.001 | 8835 | 4 |
| *Anthocaris cardamines* | Orange tip | 8.30 | -0.0055 | 0.0006 | -8.88 | <0.001 | 77601 | 446 |
| *Argynnis adippe* | High brown fritillary | 8.37 | -0.0096 | 0.0013 | -7.68 | <0.001 | 19178 | 28 |
| *Satyrium pruni* | Black hairstreak | 8.31 | 0.0145 | 0.0089 | 1.63 | 0.103 | 306 | 10 |
| *Nymphalis antiopa* | Camberwell beauty | 7.61 | 0.0425 | 0.0614 | 0.69 | 0.488 | 8 | 6 |
| *Carterocephalus palaemon* | Chequered skipper | 6.90 | - | - | - | - | 31 | 1 |

**Table S7, Sensitivity analysis of trends over time in the total abundance and species richness of bird and butterfly assemblages excluding rare species**. Rare species, which may have higher sampling errors, are defined as those occurring on fewer than 10 sites. Species assemblages are defined through the full species list ranked into quartiles by their species temperature indices (STI). Significant trends (at p < 0.05) are highlighted in bold. The overall results for CTI trends of birds (CTI-year coefficient = 0.0046 ± 0.0007, Χ^2^ = 35.1, p = <0.001) and butterflies (CTI-year coefficient = 0.0007 ± 0.002, Χ^2^ = 0.19, p = 0.66) with rare species excluded were qualitatively similar to those when they were included (main text).

| **Group** | **Assemblage (STI quartile)** | **Total abundance trend** | | | | **Species richness trend** | | | |
| --- | --- | --- | --- | --- | --- | --- | --- | --- | --- |
|  |  | Coefficient | se | z | p | Coefficient | se | z | p |
| Birds | High STI | -0.026 | 0.002 | -12.81 | **<0.001** | -0.009 | 0.002 | -4.56 | **<0.001** |
| Birds | Medium-high STI | 0.005 | 0.001 | 5.72 | **<0.001** | 0.004 | 0.001 | 4.03 | **<0.001** |
| Birds | Medium-low STI | -0.002 | 0.002 | -1.06 | 0.290 | -0.004 | 0.001 | -4.28 | **<0.001** |
| Birds | Low STI | -0.020 | 0.002 | -12.97 | **<0.001** | -0.006 | 0.001 | -4.87 | **<0.001** |
| Butterflies | High STI | 0.541 | 0.107 | 5.04 | **<0.001** | 0.003 | 0.001 | 2.30 | **0.021** |
| Butterflies | Medium-high STI | -0.003 | 0.005 | -0.46 | 0.642 | 0.002 | 0.002 | 1.35 | 0.176 |
| Butterflies | Medium-low STI | -0.006 | 0.004 | -1.70 | 0.089 | -0.001 | 0.001 | -0.60 | 0.545 |
| Butterflies | Low STI | 0.008 | 0.005 | 1.82 | 0.069 | -0.003 | 0.001 | -2.62 | 0.009 |

**Tables S8- Relationships between bird and butterfly CTI scores and land cover type extent around monitoring sites.** Models are fitted using linear mixed models with habitat extents (% cover 0.5km radius from monitoring route centroid) and site northing and easting as fixed effect explanatory variables and *Site ID*, *Year* and *50km GB grid* fitted as random effects. These latter five variables are an attempt to account for variation in CTI caused by spatiotemporal climate patterns. We fitted models for birds and butterflies using two alternative ways of defining improved and low intensity grassland (see Methods). The results tables S8a-S8h differ in the species group tested and the definition of low intensity grassland (highlighted in bold). There is one table for the coefficients in each model and one table showing correlations between land use variables.

Table S8a- Relationship between **bird** CTI scores and land cover type extent around monitoring sites using low intensity grassland defined as **lowland calcareous grassland**. Significant relationships from (at p < 0.05) are highlighted in bold.

| **Variable** | **Coefficient** | **se** | **t** |
| --- | --- | --- | --- |
| Intercept | 13.99 | 1.00E-01 | 139.9 |
| Arable | 1.39E-03 | 7.83E-04 | 1.77 |
| Broadleaved woodland | 2.04E-03 | 8.75E-04 | **2.33** |
| Improved grassland 1 | 9.35E-04 | 8.35E-04 | 1.12 |
| Low intensity grassland 1 | -1.60E-03 | 1.34E-03 | -1.19 |
| Urban/ suburban | 1.94E-03 | 7.95E-04 | **2.44** |
| Site northing | -3.65E-07 | 9.16E-08 | **-3.98** |
| Site easting | -2.16E-07 | 1.49E-07 | -1.45 |

Table S8b- Correlation between habitat types from the mixed model fitted for Table S9a. No correlations are very strong justifying the using of multiple habitat variables in the same model.

|  | Arable | Broadleaved woodland | Improved grassland 1 | Low intensity grassland 1 |
| --- | --- | --- | --- | --- |
| Broadleaved woodland | 0.72 |  |  |  |
| Improved grassland 1 | 0.61 | 0.66 |  |  |
| Low intensity grassland 1 | 0.32 | 0.36 | 0.41 |  |
| Urban/ suburban | 0.72 | 0.69 | 0.65 | 0.37 |

Table S8c- Relationship between **bird** CTI scores and habitat extent around monitoring sites using low intensity grassland defined as **lowland calcareous grassland and lowland meadows**. Significant relationships from (at p < 0.05) are highlighted in bold.

| **Variable** | **Coefficient** | **se** | **t** |
| --- | --- | --- | --- |
| Intercept | 14.04 | 1.11E-01 | 126.55 |
| Arable/ horticultural | 1.27E-03 | 8.57E-04 | 1.49 |
| Broadleaved woodland | 1.82E-03 | 9.58E-04 | 1.9 |
| Improved grassland 2 | 9.77E-04 | 9.31E-04 | 1.05 |
| Low intensity grassland 2 | -5.06E-04 | 6.22E-04 | -0.81 |
| Urban/ suburban | 1.54E-03 | 8.64E-04 | 1.78 |
| Site northing | -3.26E-07 | 1.06E-07 | **-3.06** |
| Site easting | -2.76E-07 | 1.71E-07 | -1.61 |

Table S8d- Correlation between habitat types from the mixed model fitted for Table S9c. No correlations are very strong justifying the using of multiple habitat variables in the same model.

|  | Arable | Broadleaved woodland | Improved grassland 2 | Low intensity grassland 2 |
| --- | --- | --- | --- | --- |
| Broadleaved woodland | 0.71 |  |  |  |
| Improved grassland 2 | 0.59 | 0.64 |  |  |
| Low intensity grassland 2 | 0.40 | 0.42 | 0.54 |  |
| Urban/ suburban | 0.70 | 0.68 | 0.63 | 0.45 |

Table S8e- Relationship between **butterfly** CTI scores and habitat extent around monitoring sites using low intensity grassland defined as **lowland calcareous grassland**. Significant relationships from (at p < 0.05) are highlighted in bold.

| **Variable** | **Coefficient** | **se** | **t** |
| --- | --- | --- | --- |
| Intercept | 9.40 | 9.46E-02 | 99.38 |
| Arable/ horticultural | -1.70E-05 | 7.05E-04 | -0.02 |
| Broadleaved woodland | -2.01E-03 | 7.20E-04 | **-2.79** |
| Improved grassland 1 | 9.28E-08 | 8.81E-08 | 1.05 |
| Low intensity grassland 1 | 2.50E-03 | 1.08E-03 | **2.31** |
| Urban/ suburban | 5.87E-04 | 7.77E-04 | 0.75 |
| Site northing | -1.09E-06 | 1.19E-07 | **-9.15** |
| Site easting | 4.75E-07 | 1.56E-07 | **3.05** |

Table S8f- Correlation between habitat types from the mixed model fitted for Table S9e. No correlations are very strong justifying the using of multiple habitat variables in the same model.

|  | Arable/ horticultural | Broadleaved woodland | Improved grassland 1 | Low intensity grassland 1 |
| --- | --- | --- | --- | --- |
| Broadleaved woodland | 0.69 |  |  |  |
| Improved grassland 1 | 0.59 | 0.69 |  |  |
| Low intensity grassland 1 | 0.26 | 0.34 | 0.37 |  |
| Urban/ suburban | 0.64 | 0.63 | 0.57 | 0.34 |

Table S8g- Relationship between **butterfly** CTI scores and habitat extent around monitoring sites using low intensity grassland defined as **lowland calcareous grassland and lowland meadows**. Significant relationships from (at p < 0.05) are highlighted in bold.

| **Variable** | **Coefficient** | **se** | **t** |
| --- | --- | --- | --- |
| Intercept | 9.34 | 8.70E-02 | 107.36 |
| Arable/ horticultural | 3.64E-04 | 6.28E-04 | 0.58 |
| Broadleaved woodland | -1.43E-03 | 6.40E-04 | **-2.24** |
| Improved grassland 2 | 1.70E-03 | 6.74E-04 | **2.53** |
| Low intensity grassland 2 | 1.53E-03 | 4.21E-04 | **3.64** |
| Urban/ suburban | 1.17E-03 | 7.22E-04 | 1.62 |
| Site northing | -1.10E-06 | 1.15E-07 | **-9.58** |
| Site easting | 4.81E-07 | 1.51E-07 | **3.18** |

Table S8h- Correlation between habitat types from the mixed model fitted for Table S9g. No correlations are very strong justifying the using of multiple habitat variables in the same model.

|  | Arable/ horticultural | Broadleaved woodland | Improved grassland 2 | Low intensity grassland 2 |
| --- | --- | --- | --- | --- |
| Broadleaved woodland | 0.61 |  |  |  |
| Improved grassland 2 | 0.43 | 0.58 |  |  |
| Low intensity grassland 2 | 0.39 | 0.54 | 0.69 |  |
| Urban/ suburban | 0.57 | 0.56 | 0.44 | 0.48 |

**Table S9- Comparison of model goodness of fit for different land cover characterisations.**

We selected the category of high intensity land use and spatial scale of assessment which best explained changes in the abundance of bird and butterfly low STI and high STI species assemblages**.** AICs are provided for models fitted using different groupings of high intensity land use (A = arable and horticultural, IG1 = improved grassland defined as all grasslands besides lowland calcareous grassland, IG2 = improved grassland defined as all grasslands besides lowland calcareous grassland and lowland meadows, U = urban and suburban,), at different spatial scales (km radius around monitoring site). The lowest model AICs (within 2 AIC points) for each species assemblage are highlighted in bold.

|  |  | **Butterflies** | | **Birds** | |
| --- | --- | --- | --- | --- | --- |
| Spatial scale (km) | High intensity land use classification | Low STI | High STI | Low STI | High STI |
| 0.5 | A | 287680.3 | 725925 | 16457.89 | 12462.48 |
|  | A + IG1 | 287676.7 | 725925.3 | 16458.15 | 12461.66 |
|  | A + IG2 | 287677.4 | 725920.2 | 16458.54 | 12463.99 |
|  | A + U | 287667.6 | 725925.2 | 16455.37 | 12466.98 |
|  | A + U + IG1 | 287667.4 | 725924.6 | 16458.19 | 12464.94 |
|  | A + U + IG2 | **287664.3** | **725909.8** | 16459 | 12466.35 |
| 2 | A | 287737.2 | 726880.6 | 16454.3 | **12453.5** |
|  | A + IG1 | 287743.4 | 726882.3 | 16459.0 | 12459.2 |
|  | A + IG2 | 287746.8 | 726889 | 16460.1 | 12462.2 |
|  | A + U | 287745.1 | 726889.3 | **16450.1** | 12461.6 |
|  | A + U + IG1 | 287757.9 | 726889.1 | 16457.4 | 12462.7 |
|  | A + U + IG2 | 287757 | 726886.2 | 16458.7 | 12465.5 |
| 5 | A | 287728.1 | 726880.6 | 16452.9 | **12451.7** |
|  | A + IG1 | 287721.1 | 726876.5 | 16458.7 | 12460.7 |
|  | A + IG2 | 287718.6 | 726882.3 | 16459.3 | 12461.2 |
|  | A + U | 287746.2 | 726888.9 | 16452.2 | 12459.4 |
|  | A + U + IG1 | 287741 | 726883.9 | 16457.2 | 12462.8 |
|  | A + U + IG2 | 287740.2 | 726888 | 16458.7 | 12464.1 |
| 10 | A | 287728.7 | 726882.4 | 16455.1 | 12454.7 |
|  | A + IG1 | 287713.4 | 726876.8 | 16459.5 | 12463.0 |
|  | A + IG2 | 287711.1 | 726879.4 | 16459.3 | 12463.1 |
|  | A + U | 287745.9 | 726888.1 | 16452.4 | 12457.4 |
|  | A + U + IG1 | 287726.7 | 726881 | 16456.4 | 12462.4 |
|  | A + U + IG2 | 287728.9 | 726885.1 | 16456.3 | 12463.0 |

**Table S10, Comparison of model goodness of fit for different land cover characterisations including the area of sea as hostile habitat.** We selected the category of high intensity land use and spatial scale of assessment which best explained changes in the abundance of bird and butterfly low STI and high STI species assemblages**.** AICs are provided for models fitted using different groupings of high intensity land use plus area covered by sea for coastal sites (A = arable and horticultural, S= sea, IG1 = improved grassland defined as all grasslands besides lowland calcareous grassland, IG2 = improved grassland defined as all grasslands besides lowland calcareous grassland and lowland meadows, U = urban and suburban), at different spatial scales (km radius around monitoring site). The lowest model AICs (within 2 AIC points) for each species assemblage are highlighted in bold.

|  |  | **Butterflies** | | **Birds** | |
| --- | --- | --- | --- | --- | --- |
| Spatial scale (km) | High intensity land use classification | Low STI | High STI | Low STI | High STI |
| 0.5 | A + S | 287680.5 | 725925.1 | 16457.81 | 12462.42 |
|  | A + IG1 + S | 287676.3 | 725925.4 | 16457.96 | 12461.6 |
|  | A + IG2 + S | 287677 | 725920.1 | 16458.38 | 12463.95 |
|  | A + U + S | 287667.2 | 725925.1 | 16455.33 | 12466.91 |
|  | A + U + IG1 + S | 287666.5 | 725924.6 | 16458.03 | 12464.83 |
|  | A + U + IG2 + S | **287663.6** | **725909.6** | 16458.87 | 12466.26 |
| 2.0 | A + S | 287739.1 | 726880.8 | 16454.2 | **12453.3** |
|  | A + IG1 + S | 287744.5 | 726882.3 | 16459.0 | 12459.1 |
|  | A + IG2 + S | 287747.8 | 726889.1 | 16460.1 | 12462.1 |
|  | A + U + S | 287745.2 | 726889.3 | **16449.9** | 12461.5 |
|  | A + U + IG1 + S | 287757.7 | 726889.2 | 16457.3 | 12462.6 |
|  | A + U + IG2 + S | 287756.9 | 726886 | 16458.7 | 12465.4 |
| 5.0 | A + S | 287731.8 | 726881.5 | 16452.6 | **12451.6** |
|  | A + IG1 + S | 287724.2 | 726876.9 | 16458.5 | 12460.7 |
|  | A + IG2 + S | 287721.5 | 726882.7 | 16459.2 | 12461.2 |
|  | A + U + S | 287747.2 | 726888.9 | **16451.9** | 12459.4 |
|  | A + U + IG1 + S | 287743.6 | 726883.9 | 16457.0 | 12462.7 |
|  | A + U + IG2 + S | 287742.5 | 726887.8 | 16458.5 | 12464.0 |
| 10.0 | A + S | 287730.6 | 726882.4 | 16453.9 | **12453.3** |
|  | A + IG1 + S | 287715.4 | 726876.5 | 16459.2 | 12462.7 |
|  | A + IG2 + S | 287712.9 | 726879.2 | 16458.9 | 12462.6 |
|  | A + U + S | 287746.4 | 726887.8 | **16450.8** | 12455.4 |
|  | A + U + IG1 + S | 287728.8 | 726879.7 | 16455.0 | 12460.9 |
|  | A + U + IG2 + S | 287730.8 | 726884.6 | 16454.9 | 12461.5 |

**Table S11- Model results using alternative classification of high intensity land cover or at different spatial scales.** Results are shown for models using categories of high intensity land cover and spatial scales which gave similar explanatory power based on model AIC (within an AIC difference of 2, indicating marginal differences in goodness of fit; see Tables S10 & 11). Highlighted in bold are the categorisations which gave the lowest AIC and are used for plots in the main text, but the table shows that results are qualitatively similar with the next best fitting models.

| **Species group** | **Response variable** | **Spatial scale (km)** | **High intensity land use categorisation** | **Year: area high intensity land use coefficient** | **SE** | **z** | **p** | **Year: annual temperature coefficient** | **SE** | **z** | **p** |
| --- | --- | --- | --- | --- | --- | --- | --- | --- | --- | --- | --- |
| **Birds** | **Low STI species total abundance** | **2** | **A+U** | -0.537 | 0.191 | -2.82 | <0.001 | 0.058 | 0.034 | 1.70 | 0.09 |
| Birds | Low STI species total abundance | 2 | A+U+S | -0.541 | 0.191 | -2.83 | <0.001 | 0.059 | 0.034 | 1.71 | 0.09 |
| **Birds** | **High STI species total abundance** | **2** | **A** | -0.944 | 0.262 | -3.60 | <0.001 | -0.030 | 0.042 | -0.72 | 0.47 |
| Birds | High STI species total abundance | 2 | A+S | -0.955 | 0.263 | -3.63 | <0.001 | -0.030 | 0.042 | -0.72 | 0.47 |
| Birds | High STI species total abundance | 5 | A | -1.096 | 0.281 | -3.90 | <0.001 | -0.034 | 0.042 | -0.81 | 0.42 |
| Birds | High STI species total abundance | 5 | A+S | -1.102 | 0.282 | -3.90 | <0.001 | -0.032 | 0.042 | -0.77 | 0.44 |
| Birds | High STI species total abundance | 10 | A+S | -1.124 | 0.308 | -3.65 | <0.001 | -0.031 | 0.042 | -0.75 | 0.46 |
| **Butterflies** | **Low STI species total abundance** | **0.5** | **A + U + IG2** | -0.283 | 0.091 | -3.10 | 0.002 | -0.154 | 0.010 | -14.96 | <0.001 |
| Butterflies | Low STI species total abundance | 0.5 | A + U + IG2 + S | -0.286 | 0.091 | -3.12 | 0.00 | -0.154 | 0.010 | -14.96 | <0.001 |
| **Butterflies** | **High STI species total abundance** | **0.5** | **A + U + IG2** | -0.0002 | 0.08 | 0.00 | 0.998 | 0.16 | 0.01 | 28.56 | <0.001 |
| Butterflies | High STI species total abundance | 0.5 | A + U + IG2 + S | -0.0014 | 0.08 | -0.02 | 0.99 | 0.16 | 0.01 | 28.56 | <0.001 |

**Table S12- Sensitivity analysis of interactions of bird and butterfly assemblages with land use and climate** **excluding rare species**. Interaction effects between a) area of high intensity land use and year and b) mean annual temperature and year, on the total abundance of low or high STI bird and butterfly species. Interactions effects are demonstrated by plotting abundance trends over time versus area of high intensity land use (Figure 3). Significant interactions (at p < 0.05) are highlighted in bold.

| **Species group** | **Response variable** | **Year: area high intensity LULC coefficient** | **SE** | **z** | **p** | **Year: annual temperature coefficient** | **SE** | **z** | **p** |
| --- | --- | --- | --- | --- | --- | --- | --- | --- | --- |
| Birds | Low STI species total abundance | -0.54 | 0.19 | -2.85 | **0.004** | 0.05 | 0.03 | 1.61 | 0.11 |
| Birds | High STI species total abundance | -1.03 | 0.27 | -3.80 | **<0.001** | -0.02 | 0.04 | -0.46 | 0.64 |
| Butterflies | Low STI species total abundance | -0.27 | 0.09 | -2.99 | **0.003** | -0.15 | 0.01 | -14.96 | **<0.001** |
| Butterflies | High STI species total abundance | -0.0019 | 0.07 | -0.02 | 0.98 | 0.16 | 0.01 | 28.74 | **<0.001** |

**Appendix S1- Comparison of butterfly community temperature index (CTI) trends with Devictor *et al*. .**

Devictor *et al*. showed a significant increase in UK butterfly CTI scores during 1990–2008. In contrast, the butterfly CTI trend demonstrated in this paper is not significant. Despite the difference in time periods (our data span 1976–2009), the discrepancy with Devictor *et al*. is caused by both a) the statistical methodology, and, b) the location of the monitoring sites used in the analysis. Devictor *et al*. used a mixed effects model with CTI as a response variable and *Site* as a random intercept. Fitting a similar model to the 493 UK butterfly monitoring sites that satisfied our selection criteria for time series length does result in a significant trend in CTI (Table S13; note that our model also includes a random intercept for *50km GB grid* in order to account for spatial autocorrelation, but this has negligible impact on results).

However, there is significant interannual variation in butterfly CTI scores with particular years having very high or low values (e.g. Figure 2f). Therefore we tested the inclusion of a random intercept term for *Year* (a categorical variable) which accounts for short term changes in CTI, reducing the influencing of outlying values and allows focus on long-term trends. This model has a better goodness of fit (measured by AIC score; Table S13). Additionally, the inclusion of a random slope for year (i.e. allowing trends in CTI to vary at each site) increases model goodness of fit further. These refined models are less sensitive to inter-annual variability and still detect long-term trends where they exist (e.g. for bird CTI and butterfly and bird low and high STI assemblages; Figure 2). In addition, because the models explain more variation in CTI they provide increased sensitivity to subsequently detect impacts of land use on temporal trends in CTI.

A secondary difference between our models and those of Devictor *et al*.^1^ are that we subset our data to 454 sites (92% of the total available) in England where field survey data were available, allowing the discrimination of intensive grassland. This subsetting produces different trends in CTI, even using the original Devictor *et al*.^1^ model (Table S14). The reason for this is that sites in Scotland appear to have very large trends in CTI (Table S15). Therefore, it is clear that there is large variation in CTI trends between sites within countries (Table S14) and also between countries (Table S15). Some of this variation in CTI trend between sites can be explained by land use and spatial patterns in mean annual temperature (main text results).

To summarise, in this study, we found it appropriate to use a more complex statistical model that accounted for the substantial variation in CTI between sites and between years. This is not to suggest, however, that the long-term trends in CTI found by Devictor *et al*.^1^ are erroneous. Considering the English data in Figure S1, it possible that several more years of data will confirm a positive CTI trend, whose signal can be statistically unpicked from the large interannual variability.

**Table S13- Models with varying random effect structures for assessing temporal trends in butterfly community temperature index (CTI) across the UK**

| **Region** | **Number of sites** | **Model structure** | **Coefficient** | **se** | **t** | **p** | **AIC** |
| --- | --- | --- | --- | --- | --- | --- | --- |
| UK | 493 | CTI ~ year + (1|site) + (1|50kmGrid) | 0.0009 | 0.0004 | 2.39 | 0.017 | -2468 |
| UK | 493 | CTI ~ year + (1|site) + (1|year)+ (1|50kmGrid) | -0.0001 | 0.0014 | -0.07 | 0.945 | -3164 |
| UK | 493 | CTI ~ year + (year|site) + (1|year)+ (1|50kmGrid) | 0.0005 | 0.0014 | 0.34 | 1.000 | -3250 |
|  |  |  |  |  |  |  |  |

**Table S14- Models with varying random effect structures for assessing temporal trends in butterfly community temperature index (CTI) in England**

| **Region** | **Number of sites** | **Model structure** | **Coefficient** | **se** | **t** | **p** | **AIC** |
| --- | --- | --- | --- | --- | --- | --- | --- |
| England | 454 | CTI ~ year + (1|site)+ (1|50kmGrid) | 0.0006 | 0.0004 | 1.52 | 0.130 | -2525 |
| England | 454 | CTI ~ year + (1|site) + (1|year)+ (1|50kmGrid) | -0.0009 | 0.0015 | -0.59 | 0.556 | -3222 |
| England | 454 | CTI ~ year + (year|site) + (1|year)+ (1|50kmGrid) | -0.0080 | 0.0010 | -0.58 | 0.582 | -3945 |

**Table S15- Models assessing temporal trends in butterfly community temperature index (CTI) in three UK countries**

| **Region** | **Number of sites** | **Model structure** | **Coefficient** | **se** | **t** | **p** |
| --- | --- | --- | --- | --- | --- | --- |
| England | 454 | CTI ~ year + (1|site) + (1|50kmGrid) | 0.0006 | 0.0004 | 1.52 | 0.130 |
| Scotland | 22 | CTI ~ year + (1|site) + (1|50kmGrid) | 0.0068 | 0.0023 | 2.93 | 0.004 |
| Wales | 16 | CTI ~ year + (1|site) + (1|50kmGrid) | 0.0098 | 0.0130 | 0.75 | 0.458 |
